# Differential apoptotic response of MC3T3-E1 pre-osteoblasts to biodegradable magnesium alloys in an in vitro direct culture model

**DOI:** 10.1007/s10856-017-5969-5

**Published:** 2017-09-05

**Authors:** Ehsan Bonyadi Rad, Sepideh Mostofi, Matthias Katschnig, Patrik Schmutz, Magdalena Pawelkiewicz, Regine Willumeit-Römer, Ute Schäfer, Annelie Weinberg

**Affiliations:** 1 Department of Orthopedics and Trauma Surgery, Medical University Graz, Graz, Austria; 2 Department of Polymer Engineering and Science, Montanuniversitaet Leoben, Leoben, Austria; 3 EMPA, Swiss Federal Laboratories for Materials Science and Technology, Duebendorf, Switzerland; 4 Institute of Materials Research, Division Metallic Biomaterials, Helmholtz-Zentrum Geesthacht, Geesthacht, Germany; 5 Department of Neurosurgery, Medical University Graz, Graz, Austria

## Abstract

**Abstract:**

The biodegradable magnesium-based implants have been widely utilized in medical orthopedic applications in recent years. We have recently shown that direct culture on Pure Mg and Mg2Ag alloys lead to a progressive differentiation impairment of MC3T3-E1 pre-osteoblasts. In this study, we aimed to analyze the apoptotic reaction of MC3T3-E1 cells in response to the direct culture on Pure Mg, Mg2Ag and Extreme High Pure Mg (XHP Mg) alloy samples. Our results demonstrated that long-term culturing of MC3T3-E1 cells on Pure Mg and Mg2Ag alloys induce time-dependent expression of active caspase-3 (active casp-3) and cleaved PARP-1 (cl. PARP-1), the hallmark of apoptosis reactions concomitant with a significant increase in the number of dead cells. However, direct culture on XHP Mg material results in a lower number of dead cells in comparison to Pure Mg and Mg2Ag alloys. Furthermore, XHP Mg materials influence expression of apoptotic markers in a process resembles that of observed in osteogenic condition apparently indicative of MC3T3-E1 osteodifferentiation. This study indicates that Mg alloy samples mediated differential apoptotic reactions of MC3T3-E1 cells can be ascribed to factors such as distinct topography and hydrophobicity features of Mg material surfaces, contrasting nature/composition of corrosion products as well as different impurities of these materials. Therefore, initial Mg alloys surface preparation, controlling the growth and composition of corrosion products and Mg alloys purity enhancement are necessary steps towards optimizing the Mg alloys usage in medical orthopedic applications.

**Graphical Abstract:**

## Introduction

In recent years, biodegradable magnesium-based implants have attracted extensive interests in orthopedic applications due to financial and clinical advantages over conventional non-resorbable bio-inert metal and plastic implants. Biodegradation characteristics, inherent biocompatibility, high specific strength, load-bearing features and elasticity resembling natural bones are suggested as some of the important benefits associated with biodegradable magnesium-based implants utilization in medical orthopedic applications [1–3]. Although investigations have recorded several advantages, the limitations of Mg implants mainly associated with low corrosion resistance are reported as well which confine Mg-based implant usage in orthopedic applications [2, 4–6]. In vivo Mg biomaterial implantation might trigger the normal and harmless foreign body associated immunological responses [7]. An essential supporting mechanism is, therefore, apoptosis a system required to remove the inflamed and injured tissues necessary for regeneration and healing processes [8]. Apoptosis or programmed cell death can commonly take place as a homeostatic factor to maintain the cell populations during development and aging or as a defense mechanism in immune reactions or when cells are damaged by disease or harmful agents [9]. Caspases the family of cysteine proteases are key mediators of apoptosis pathway [10–12]. One of the most important executioners of apoptosis in this family is caspase-3 [13] which normally exists in the cells in an inactive form [14]. In response to the apoptotic signals inactive form of caspase-3 is cleaved to the active form (active casp-3) by initiator caspases [9, 13]. Activation of caspase-3 (and other apoptosis executioner members like caspase-6 or caspase-7) leads to the cleavage of several substrates including poly [ADP-ribose] polymerase (PARP), resulting eventually in cellular and morphological variations, typically observed in apoptotic cells [9].

Our recent in vitro study [15] has demonstrated the optimal biocompatibility of non-corroded (without pre-incubation) Mg10Gd materials with murine pre-osteoblastic MC3T3-E1 cells in a long term direct culture which resulted in re-induction of osteoblast differentiation markers. On the contrary, progressive differentiation impairment of MC3T3-E1 cells on Pure Mg and Mg2Ag alloy samples were documented. Based on these observations, in this study, we aimed to analyze the effect of non-corroded Pure Mg, Mg2Ag and XHP Mg (enhanced purity) alloy samples on the apoptotic response and survival of MC3T3-E1 cells in a direct incubation fashion. Our investigation suggests the pivotal role of Mg samples surface characteristics on the apoptotic/viability response of the MC3T3-E1 cells in a direct in vitro culture system.

## Materials and methods

### Cell culture

Murine pre-osteoblast MC3T3-E1 cells were obtained from European collection of cell cultures (ECACC, Salisbury, UK). Eagle’s minimum essential medium (Sigma-Aldrich, Vienna, Austria) supplemented with 10% fetal bovine serum (Sigma-Aldrich), penicillin (100 U ml^−1^), streptomycin (100 µg ml^−1^) and 2 mM glutamine (Invitrogen, CA, USA) were used as growth media. For direct culture, 5 × 10^4^ MC3T3-E1 cells were seeded on untreated Pure Mg, Mg2Ag and XHP-Mg in growth media for 2, 6 and 12 days. For cell harvest, after washing with PBS the cells were incubated with 2 mL of Accutase (Invitrogen) for 10 minutes under cell culture condition. Collected cells were then centrifuged and stored in −20 °C for further analysis. In osteogenic differentiation condition, 50 µg/ml ascorbic acid and 10 mM beta-glycerophosphate were added to the growth media. The cells were maintained at 37 °C, in a humidified atmosphere (95%) with 5% CO2. The media changed every other day in all conditions.

### Live/dead staining

Live/dead staining was performed according to previous report [15]. The Cell viability was determined using Live/Dead staining (Invitrogen). The culture medium was eliminated from 2, 6 and 12 days cells cultured on untreated Mg and Mg alloys and then samples were washed with phosphate buffer solution (PBS, Gibco, Invitrogen). After washing step the samples were incubated with 10 mL PBS containing 5 µl calcein AM and 20 µl ethidium homodimer-1 for 30 minutes at room temperature in a dark condition. Subsequently, samples were washed twice using CaCl2 and MgCl2-free phosphate buffered saline (PBS, Invitrogen) and pictures were taken by inverted fluorescence  microscope (Olympus).

### Immunoblotting

Immunoblotting was performed according to the previous studies [15, 16]. The immunoblotting was performed with whole cell lysates generated by RIPA buffer (Sigma Aldrich). Bradford protein assay (BioRad, Hercules, CA, USA) were used to determine the protein concentration of samples. 5 μg proteins per lane were loaded and separated on 10% SDS-polyacrylamide gel. The gel was transferred to a polyvinylidene difluoride (PVDF) membrane and probed with specific primary antibodies. Signals were detected after incubation with peroxidase-conjugated secondary antibody. Anti PARP-1 (46D11), cleaved PARP-1 (D214) and cleaved/active caspase3 (D175) primary antibodies were purchased from Cell Signaling. Anti β-Actin antibody (N-21) and secondary antibodies were bought from Santa Cruz. Protein bands were visualized using ECL reagents (Amersham, little Chalfont, UK) and exposed to X-ray film. Before probing with primary antibodies, the membranes were cut to avoid stripping.

### Materials and sample preparation

The Pure Mg and Mg2Ag material preparations were described previously [15]. The Extreme High Pure Mg (XHP) material was produced via a vacuum distillation purification process. Samples were produced from a distilled block of XHP-Mg in a cuboidal shape with a cross-section of 10 × 10 mm^2^ and a thickness of 2 mm. The sample surfaces were ground manually. Grinding was performed by abrasive SiC paper of granularities 2500 and 4000 in deionized water. Subsequently, the specimens were cleaned in a cascade of acetone in an ultrasonic bath and dried with hot air in medical grade clean room atmosphere (class 100,000). The samples were packaged air-tight and sterilized using gamma irradiation (25 kGy). Table 1 illustrates the composition of alloying elements of the Mg materials used in this study.

**Table 1:** Composition of magnesium based materials used in this study [15]

	Composition Wt.%				
Alloy	Fe	Cu	Ni	Ag	Mg
Pure Mg	0.0055	0.003	0.0018	–	Bal.
Mg2Ag	0.0022	0.002	0.0013	1.75	Bal.
Xhp-Mg	0.00002	0.0001	0.0001	–	Bal.

### Atomic force microscopy (AFM)

The AFM micrographs were recorded with a Nanosurf Flex AFM instrument (Nanosurf, CH) and silicon AFM probes with a resonance frequency of 1.3 kHz and a force constant of 0.2 N/m (ContAL-G, Budgetsensors). Scan area was 80 × 80 µm and evaluated area was 60 × 60 µm to avoid fringe effects. Data processing was conducted with Gwyddion Version 2.44 (Czech Metrology Institute, Czech Rep.).

### Contact angle measurements

A 2 µl distilled deionized water droplet was positioned on the Mg samples and images were captured to measure the angle formed at the liquid-solid interface (static sessile drop method) on each Mg alloys surface. The Drop Shape Analyzer DSA100S (Kruess GmbH, Hamburg, Germany) was used for this test. Mean values were calculated from at least six individual measurements. Reproducibility was assured by a maximum standard deviation of ±2°.

### Scanning electron microscopy (SEM/EDX)

The Mg samples were analyzed after 12 days of cell incubation by scanning electron microscope equipped with Energy-dispersive X-ray spectroscopy (Hitachi-S3700N). Local area and map distribution of the elements in the corrosion products were analyzed by EDX. For the cross section analysis of the corrosion products, HITACHI-M4000 ion milling system was used. The intended cross sectional cutting edge is defined by the sharp edge of a mask placed onto the surface of the sample. The part of the sample that extends out from the edge of the mask (shielding plate)–typically a few 10 µm—was then subjected to be sputter/etched by the incident Ar ions. A flat cross sectional surface was generated vertically below the mask edge. This method provides the highest precision for milling of laterally broad domains (mm range) and is ideal for subsequent high- resolution imaging/analysis without damaging (mechanically, chemically) the sample surface.

### Statistical analysis

The SPSS software (SPSS Inc., Chicago, IL, USA) was used for statistical analysis. The differences between mean values of quantitative data were analyzed by One-way ANOVA test. Statistical significance was set at 0.05 and 0.01.

## Results

### Elevated number of dead cells on Pure Mg and Mg2Ag but Not on XHP Mg samples

**Fig. 1:**
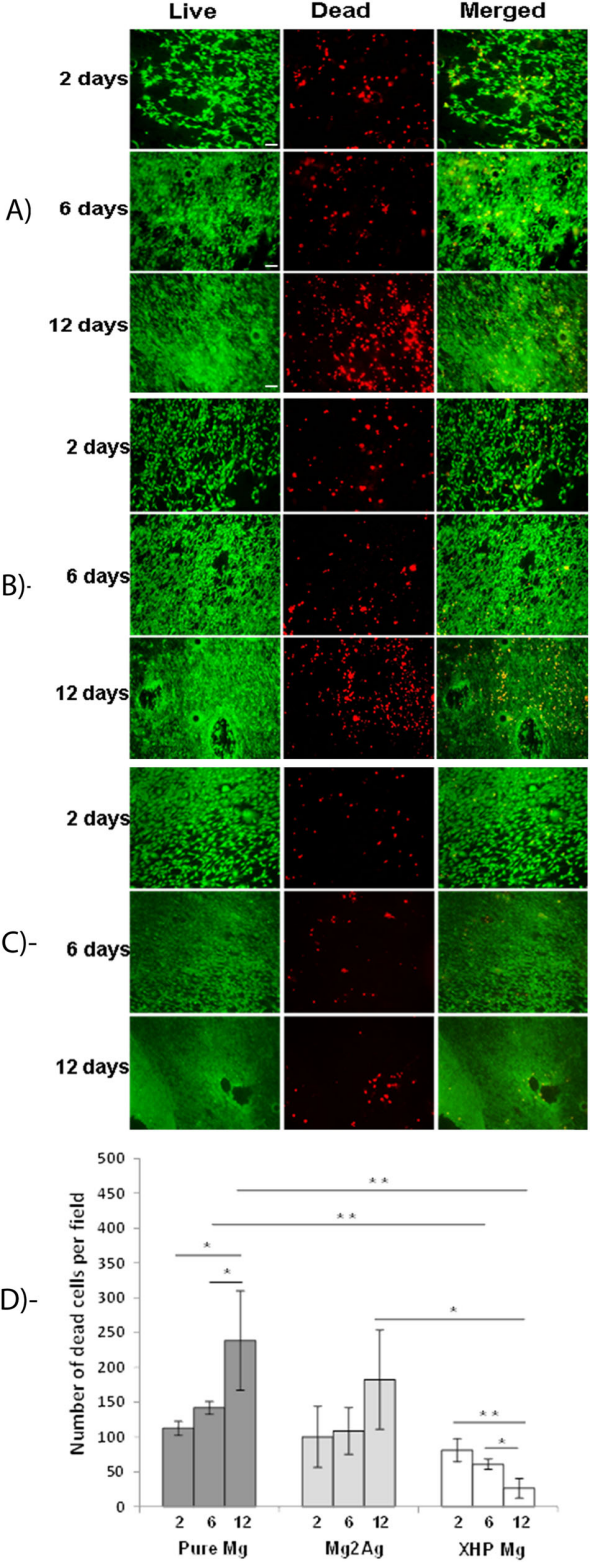
Representative pictures of live/dead assessment (left panels live cells, middle panels dead cells and right panels merged pictures) of MC3T3-E1 pre-osteoblasts cultured on non-corroded Pure Mg **a**, Mg2Ag **b** and XHP Mg **c** samples for 2, 6 and 12 days. Magnification of 10X with scale bar of 100 µm for each picture is shown. **d** Quantification of cell mortality after 2, 6 and 12 days of direct culture on Pure Mg, Mg2Ag and XHP Mg materials determined by counting the dead cells. Each experiment repeated three times. Statistical significance are * *P* < 0.05, ** *P* < 0.01

Our published data [15] showed high viability in long-term culture (12 days) of MC3T3-E1 cells on non-corroded Pure Mg, Mg2Ag and Mg10Gd alloys. However, this result was accompanied by gradual reduction of the expression of osteogenic markers Col I and Runx2 in cells grown directly on Pure Mg and Mg2Ag alloys but not on Mg10Gd alloy samples. In order to accurately evaluate the impact of Mg materials on the viability of MC3T3-E1 pre-osteoblasts, cells were cultured directly on non-corroded Pure Mg, Mg2Ag and XHP Mg alloys for 12 days and subjected to live/dead quantification in different time intervals. Even though noticeable viability was retained in all time points in all 3 types of Mg materials an increasing number of dead cells were observed during 12 days of direct culture on Pure Mg and Mg2Ag alloys (Fig. 1a, b). In contrast, culture on XHP Mg resulted in a decreasing number of dead cells during 12 days of incubation opposite to the cell response observed in Pure Mg and Mg2Ag samples (Fig. 1c). The quantifications demonstrated the evident difference between the number of dead cells present on Pure Mg and Mg2Ag samples compared to XHP Mg materials at different time points. The most significant difference was though observed at day 12. In fact, whereas 12 days of direct culture on Pure Mg led to the highest number of dead cells among others, cells showed the optimum reactions with minimum cell death number after 12 days of direct culture on XHP Mg samples (Fig. 1d). These results revealed time dependent promotion of cell mortality triggered by culturing on pure Mg and Mg2Ag but not XHP Mg alloy samples that indicate differential MC3T3-E1 reactions to Mg-based materials.

### Non-corroded Mg alloy samples differentially induce expression of apoptotic markers

**Fig. 2:**
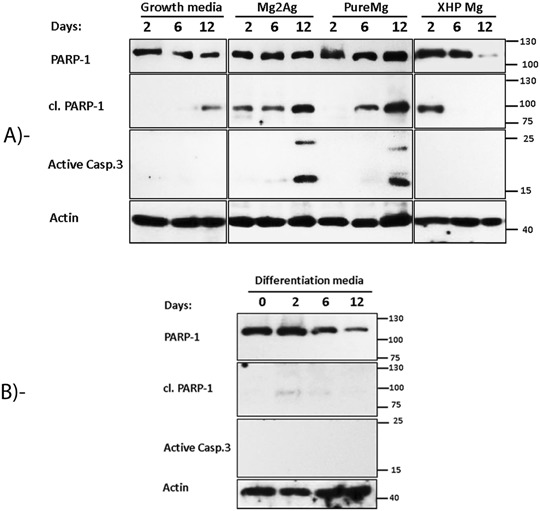
Representative Immunoblotting of the expression level of PARP-1, cl.PARP-1 and active casp-3 after 2, 6 and 12 days of MC3T3-E1 culture on 48-well tissue culture plates, Pure Mg, Mg2Ag and XHP Mg alloy samples **a** or at day 0, 2, 6 and 12 in osteogenic condition in 48 well tissue culture plates **b**. B-actin was used as a loading control

Immunoblot assessment indicated a gradual increase in active casp-3 expression, the key executioner of apoptosis on day 6 reaching to the peak expression on day 12 when cells grown on Pure Mg materials (Fig. 2a). A similar pattern of active casp-3 expression was observed from the cells cultured on Mg2Ag materials. To scrutinize the effect of cell confluence on apoptotic induction of MC3T3-E1, cells cultured on tissue culture plates (polystyrene) were used as a control. The immunoblotting showed no activation of casp-3 in these control cells during 12 days of culture. The nuclear enzyme PARP-1 which functions in repair mechanism of DNA damage has been shown as a common cellular target of active casp-3 during apoptosis [17, 18]. As compared to the control cells with the low expression level of cl. PARP-1 on day 12, cell incubation on both pure Mg and Mg2Ag alloys resulted in a progressive generation of cl. PARP-1. The highest expression level of cl. PARP-1 was observed on day 12 corresponding to the peak expression level of active casp-3. Interestingly, 12 days of cell incubation on XHP Mg did not lead to the expression of active casp-3 at any time points. In addition, the expression pattern of cl. PARP-1 in these cells was inconsistent to those observed for Pure Mg and Mg2Ag alloys. Surprisingly, cl. PARP-1 expression appeared at day two and suppressed on days 6 and 12. Moreover, whereas our results revealed the progressive suppression of enzyme PARP-1 in cells cultivated on XHP Mg alloys, no noticeable variation in expression level of this enzyme was observed in cells incubated on Pure Mg and Mg2Ag materials. Overall, our in vitro results suggest that long-term culturing of MC3T3-E1 cells lead to the differential apoptotic response dependent to the Mg samples (Fig. 2a).

Non-apoptotic role of caspases and PARP-1 were reported before [17, 19–21]. To assess whether expression pattern of active casp-3, PARP-1 and cl. PARP-1 is a non-apoptotic /differentiation dependent process, we analyzed expression of these markers during 12 days course of MC3T3-E1 differentiation (Fig. 2b). Immunoblotting represented no expression of active casp-3 during 12 days of MC3T3-E1 culture in differentiation media. Interestingly, low protein expression of cl. PARP-1 was observed at initial two days of culture in osteogenic condition. This expression was down-regulated at later time points resulting in a negligible expression at day 12. Furthermore, expression of enzyme PARP-1 decreased during osteodifferentiation period giving rise to a similar expression pattern observed for cl. PARP-1. Expression pattern of these markers in osteogenic condition resemble that observed in XHP Mg alloy samples suggesting presumably an ongoing osteodifferentiation process triggered by cell cultured on the XHP Mg samples. On the contrary, expression pattern of apoptosis markers in osteogenic condition was inconsistent to Pure Mg and Mg2Ag alloys indicating that Pure Mg and Mg2Ag alloys based activation of casp-3 and cl. PARP-1 are independent of osteodifferentiation but associated truly to the apoptosis process.

### AFM analysis of Mg alloys revealed pronounced differences in surface topography

**Fig. 3:**
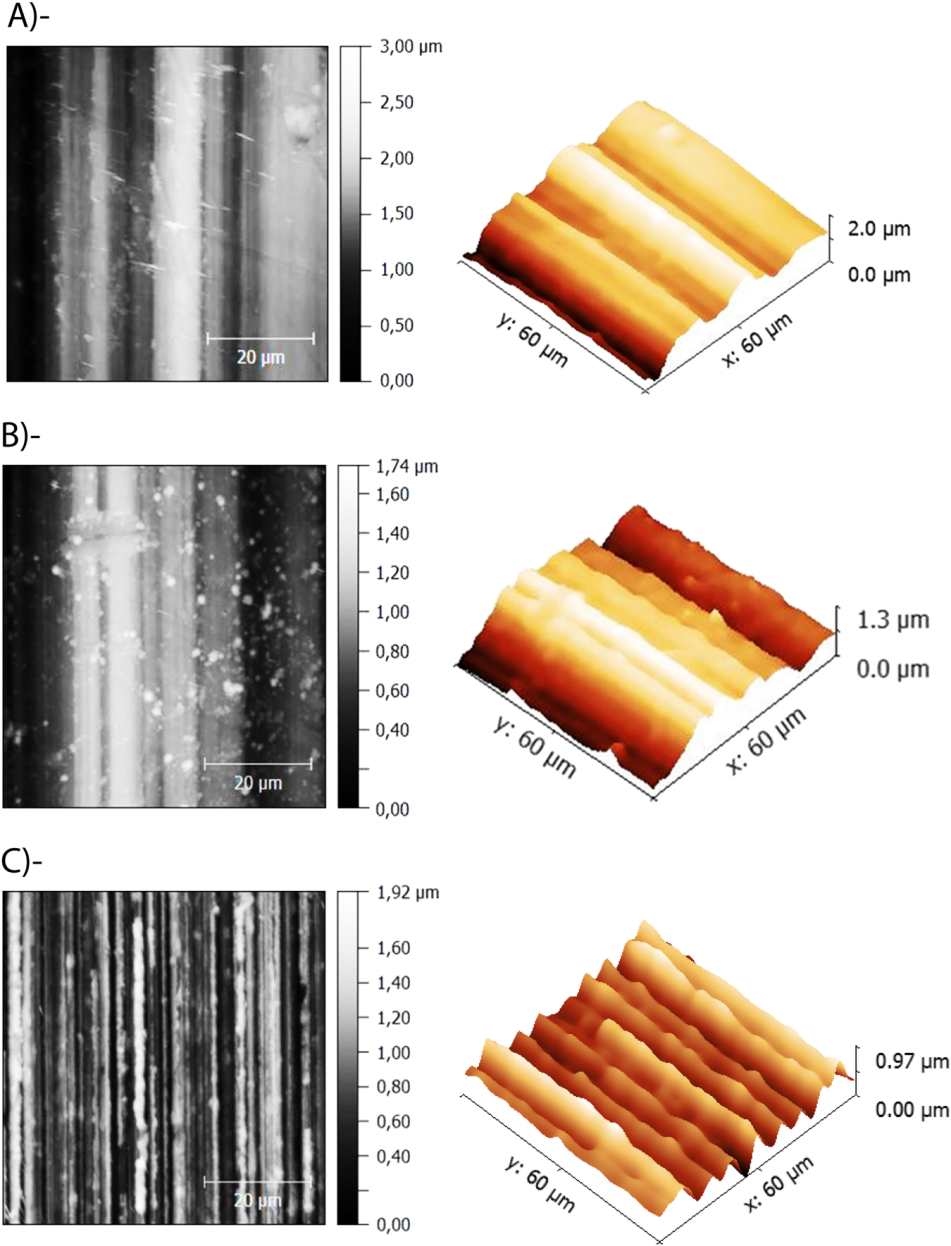
Surface topography of Pure Mg **a**, Mg2Ag **a** and XHP Mg **c** determined by Atomic Force Microscopy. Left: 2D (top view), Right: 3D

The evaluation of surface topography of Pure Mg, Mg2Ag and XHP Mg alloys prior to cell incubation using AFM revealed structural differences in topographical features (Fig. 3). A grooved structure was found in all samples. The main difference between the alloy surfaces was the number of structure elements (grooves/pits) per area unit (each 60 × 60 µm area was designated as one area unit). The AFM assessment indicated XHP Mg alloys to possess the highest number of grooves/pits in each unit compared the other two materials. The average width of a groove on XHP Mg was 7 µm, whereas the average width on Pure Mg and Mg2Ag were 30 µm and 50 µm respectively. Moreover, the highest surface point (amplitude) among three alloys was measured 3 µm for Pure Mg in one area unit compared to 1.74 and 1.92 µm measured for Mg2Ag and XHP Mg alloy samples respectively (Fig. 3).

### Contact angle measurements of Mg alloys indicated difference in surface hydrophobicity

**Fig. 4:**
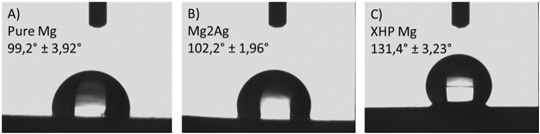
Water contact angles on the Pure Mg **a**, Mg2Ag **b** and XHP Mg **c** determined by contact angle measurements (static sessile drop)

The measurements of water contact angles yielded 99.2°, 102.2° and 131.4° on Pure Mg, Mg2Ag and XHP Mg alloy samples respectively (Fig. 4a–c). This implies hydrophobic behavior of all three material surfaces. However, while Pure Mg and Mg2Ag material surfaces showed notable similarity in wettability feature, the XHP Mg samples possesse the least wettability demonstrating the highest hydrophobic characteristics among others.

### SEM/EDX investigation revealed pronounced different corrosion products composition

**Fig. 5:**
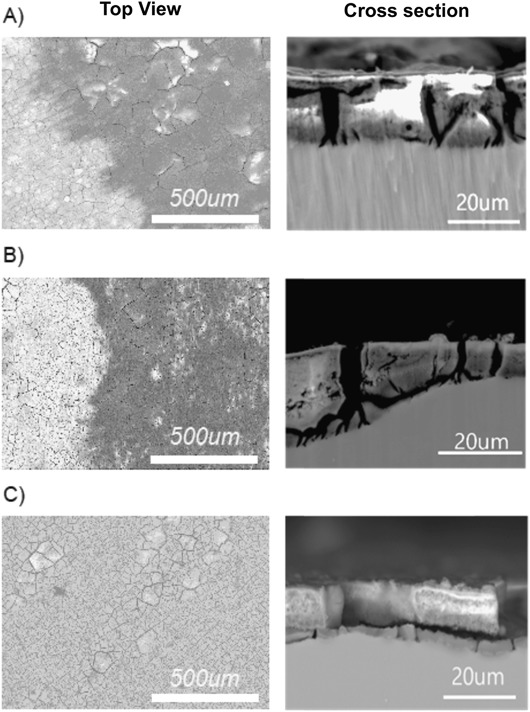
SEM characterization (Top view left and cross section right) of corrosion products on the pure Mg **a**, Mg2Ag **b** and XHP Mg **c** alloys after 12 days of cell incubation. Magnifications are 100× for top view and 2000× for cross section pictures

**Fig. 6:**
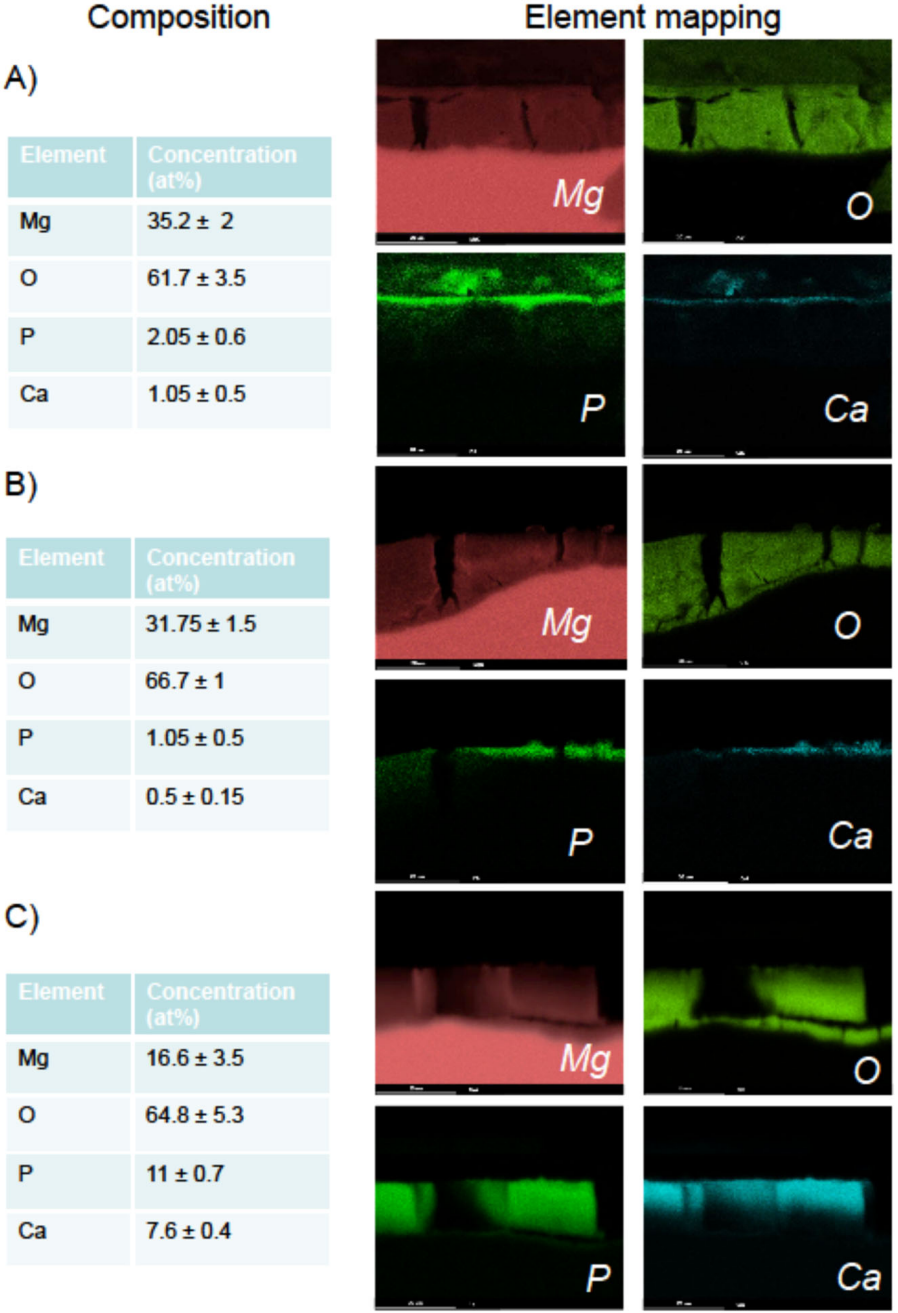
SEM/EDX mappings of the Ion beam cross-sectioned corrosion products for the pure Mg **a**, Mg2Ag **b** and XHP Mg **c** materials. Concentration values calculated in atomic percentage (at %). Magnification of 2000× with scale bar of 20 µm is shown

To characterize the corrosion product formation/composition induced by cell and culture media interaction, SEM was performed after 12 days of cell incubation. The surface topography was first imaged and similar cracked microstructures were visualized in all three types of samples (Fig. 5 a–c left panels). Moreover, no crystal formation was observed on the material surfaces. A difference observed by SEM was the presence of large black areas (around 50% of the surface) on pure Mg and Mg2Ag, while the XHP Mg surface was free of black area (Fig. 5 a–c left panels). The EDX analysis from the surface revealed that the black areas were covered with significant amount of carbon (around 60–80% of the measured composition). The rest of the surface showed 10–15% carbon which is typical for samples exposed to the air. The cross sectional SEM images (Fig. 5a, c, right panels) indicated presence of 17 to 20 and 13 to 16 µm corrosion products on Pure Mg and XHP Mg samples respectively. The thickness of the corrosion product layer formed on the XHP Mg surface was slightly thinner than that measured for Pure Mg materials. The ﻿thickness of﻿﻿ Mg2Ag corrosion product layer was however highly variable ranging 8 to 25 µm. Furthermore, the cross-sectional evaluation by EDX, displayed noticeable difference in the composition of corrosion products obtained from a series of point analysis (Fig. 6). 6 different areas on each Mg sample were evaluated. Whereas the corrosion products of the XHP Mg contained substantial amount of P (~11%) and Ca (~7.5%), Pure Mg and Mg2Ag corrosion products were mostly consist of Mg hydroxide (Mg (OH)_2_) (Fig. 6a–c). Moreover, similar percentage of Mg (OH)_2_ in pure Mg and Mg2Ag corrosion products, and a low percentage of Ca and P on these samples were observed (Fig. 6). The Mg/O concentration ratio of XHP Mg was low due to the presence of phosphates (P–O bonds) in the corrosion products of this material. Element mappings of the cross sections were also performed for the areas in the SEM images of the Fig. 5. The mapping of the in-depth distribution of the main elements obtained by EDX exhibited uniform distribution of the P and Ca throughout the corrosion products of the XHP Mg sample (Fig. 6). However, in the Pure Mg and Mg2Ag materials, slight enrichment in P and Ca concentration was observed only on the surface of the corrosion products correlated with the presence of the rich layer of carbon.

## Discussion

In the present study, it could be demonstrated that long-term incubation of MC3T3-E1 cells on Pure Mg and Mg2Ag alloy samples lead to the time dependent induction of apoptosis/cell death while incubation of MC3T3-E1 on XHP Mg give rise to the cell reactions analogous to osteodifferentiation process. Numbers of biocompatibility and cytotoxicity tests performed so far investigated cell viability of short-term and long-term incubation of the cells on the materials. For instance, Da-Tren Chou et. al, reported relatively few apoptotic MC3T3-E1 cells compared to living cells after 3 days of culture on Mg–Y–Ca–Zr alloys suggesting good cell viability. However, this viability was shown to be lower than cells on tissue culture plastics [22]. Our quantitative measurement of the cell mortality by live/dead staining clearly displayed a time-dependent rise in the number of dead cells directly cultured on Pure Mg and Mg2Ag alloy samples but not on the XHP Mg samples after 12 days of culture. It should be noted though, that the qualitative assessment of cell proliferation showed pronounced proliferation rate in all three types of Mg materials suggesting generally good cell viability at different incubation time points. However, increased incubation time was not favorable for the cells grown on Pure Mg and Mg2Ag materials contrary to the cell reactions to XHP Mg culture. Consistent with cell death number, immunoblotting demonstrated gradual and progressive increase in the expression of main apoptotic markers, active casp-3 and cl. PARP-1 in cells reacted to Pure Mg and Mg2Ag culture. On the other hand, lack of active casp-3 expression alongside diminished expression of PARP-1 and cl. PARP-1 in longer incubation times by XHP Mg culture was similar to that in osteogenic condition. Therefore, time dependent reduction of these markers can be suggestive of continuous MC3T3-E1 osteodifferentiation process mediated by XHP Mg culture. This conclusion however should be verified by further experimental examination of specific osteoblast differentiation markers.

The deleterious effect of biodegradable Mg implants can be associated mainly with the factors like low corrosion resistance, changes in the PH values and osmolality, hydrogen gas formation and material compositions [2, 4–6, 23]. Furthermore, constituents of the medium during in vitro testing would be another factor influencing corrosion rate [24] and thus might affect different cell reactions to the materials. The Mg alloys corrosion is principally accompanied with enhanced PH and ion release [25, 26] and as discussed in previous reports [4–6], severe PH (alkalization) and osmolality disturbance can adversely affect cell survival leading to increased cell death. The viability evaluation of the MC3T3-E1 cells demonstrated that increased PH up to maximum 8.6 induced by corrosion of Pure Mg and Mg2Ag samples (extracts) did not affect the viability rate [15]. Consistent with this report, only PH values above 8.6 were reported to adversely impact MC3T3-E1 cell viability [5]. Therefore, since we indicated that PH remained relatively constant varying between 8.5 and 8.6 [15], the detrimental effect of corrosion mediated PH/osmolality changes on the apoptosis induction can be excluded during Pure Mg and Mg2Ag materials degradation course. Furthermore, the evaluation of the Pure Mg and Mg2Ag corrosion profiles showed increase in the Mg ion release into the supernatant only during initial days of Mg alloys degradation that plateaued in the later time points [15] thus suggesting likely a minimal corrosion/degradation impact on the apoptotic/viability of the cells in the late incubation time points.

Mg alloys surface characteristics and morphologies are crucial factors impacting cellular metabolisms. The AFM results have clearly demonstrated the substantial difference in topographical features of Mg alloy samples with the maximum number of grooves/pits on the XHP Mg surface compared to other Mg materials. More importantly, the contact angle measurement experiment indicated XHP Mg samples to have the most hydrophobic surfaces. Interestingly, a report studied the impact of biomaterial surface chemistry on the apoptosis activation of adherent macrophages demonstrated that hydrophilic surfaces cause a lower level of adhesion along with significant apoptosis induction in comparison to the hydrophobic surfaces [27]. In addition, Mg alloys with superhydrophobic surface show remarkable corrosion resistant effect [28]. In line with these studies, we observed the lowest rate of apoptosis/dead cells induced by XHP Mg samples possessing extreme hydrophobic surface properties. Therefore, enhanced hydrophobic nature of the XHP Mg surfaces alongside grooved surface topography and potential strengthened corrosion resistance appears to be crucial factors leading to the higher level of cell adhesion and thus decreased number of apoptotic/dead cells especially in the late incubation time points. The SEM/EDX analysis further pointed out distinct corrosion product nature of the XHP Mg with a large amount of P and Ca. On the other hand, Pure Mg and Mg2Ag corrosion products contained mainly Mg (OH)_2_. At this point, it remains to be clarified that to which extent the distinct composition of corrosion products would play a role in cell viability/apoptosis or differentiation of the cells. Furthermore, large amounts of P and Ca mostly in the corrosion products of the XHP Mg material with greater extent of hydrophobicity and distinct surface morphology needs to be further elucidated from the cell and culture media interaction perspective.

It should be also taken into account that the impurities and alloy compositions [23] might play a role in differential apoptotic and cell viability responses. The toxic nature of Ag element which might result in cell viability reduction and apoptosis induction [29, 30] however, can be excluded considering no significant difference in dead cell number between Pure Mg and Mg2Ag materials. In addition, apoptotic markers are similarly induced by these two materials indicating that Ag is not a crucial factor in different apoptotic responses. The quantities of trace elements Cu, Fe and Ni present in Pure Mg and Mg2Ag alloy samples are, however, higher than that in XHP Mg materials showing the higher purity of XHP Mg alloys. Therefore, the cytotoxic effect of the Mg material impurities like Cu and Ni [31] with their low solid-solubility limits [32] might be also another contributing factor in MC3T3-E1 differential apoptotic responses to Pure Mg and Mg2Ag alloy samples in comparison with XHP Mg materials.

## Conclusions

Overall, differential viability/apoptotic response of the pre-osteoblast MC3T3-E1 cells was observed after direct incubation with various Mg alloys. The inconsistency between cell death number and expression pattern of apoptotic markers observed on Pure Mg and Mg2AG samples compared to XHP Mg alloys (considering the same in vitro culture conditions) can be generally ascribed to the topographical differences of the material surfaces, diverse hydrophobicity nature of Mg alloys surfaces, and likely presence of trace impurities and the corrosion product growth/compositions. Therefore, Initial magnesium alloys preparation procedures including refinement/tuning of the materials surface morphology and hydrophobic/hydrophilic properties, purity enhancement of Mg alloys and controlling the growth/composition of corrosion products (biodegradation) can be good strategic approaches towards optimizing the Mg alloys usage in medical orthopedic applications.
